# Relative adrenal insufficiency is a risk factor and endotype of sepsis - A proof-of-concept study to support a precision medicine approach to guide glucocorticoid therapy for sepsis

**DOI:** 10.3389/fimmu.2022.1110516

**Published:** 2023-01-12

**Authors:** Chia-Hua Wu, Ling Guo, Dan Hao, Qian Wang, Xiang Ye, Misa Ito, Bin Huang, Chieko Mineo, Philip W. Shaul, Xiang-An Li

**Affiliations:** ^1^ Department of Pharmacology and Nutritional Sciences, University of Kentucky College of Medicine, Lexington, KY, United States; ^2^ Saha Cardiovascular Research Center, University of Kentucky College of Medicine, Lexington, KY, United States; ^3^ Division of Cancer Biostatistics, Department of Internal Medicine, University of Kentucky College of Medicine, Lexington, KY, United States; ^4^ Department of Pediatrics, University of Texas Southwestern Medical Center, Dallas, TX, United States; ^5^ Lexington Veterans Affairs (VA), Healthcare System, Lexington, KY, United States; ^6^ Department of Physiology, University of Kentucky College of Medicine, Lexington, KY, United States

**Keywords:** adrenal insufficiency, corticosteroid therapy, relative adrenal insufficiency, scavenger receptor BI, sepsis

## Abstract

**Introduction:**

25-60% of septic patients experience relative adrenal insufficiency (RAI) and glucocorticoid (GC) is frequently used in septic patients. However, the efficacy of GC therapy and whether GC therapy should be based on the status of RAI are highly controversial. Our poor understanding about the pathogenesis of RAI and a lack of RAI animal model present significant barriers to address these critical issues.

**Methods:**

Scavenger receptor BI (SR-BI) regulates stress-induced GC (iGC) production in response to stress. We generated SF1CreSR-BIfl/fl mice and utilized the mice as a RAI model to elucidate the pathogenesis of RAI and GC therapy in sepsis. SF1CreSR-BIfl/fl mice did not express SR-BI in adrenal gland and lacked iGC production upon ACTH stimulation, thus, they are RAI.

**Results and Discussion:**

RAI mice were susceptible to cecal ligation and puncture (CLP)-induced sepsis (6.7% survival in SF1CreSR-BIfl/fl mice versus 86.4% in SR-BIfl/fl mice; p = 0.0001). Compared to a well-controlled systemic inflammatory response in SR-BIfl/fl mice, SF1CreSR-BIfl/fl mice featured a persistent hyperinflammatory response. Supplementation of a low stress dose of GC to SF1CreSR-BIfl/fl mice kept the inflammatory response under control and rescued the mice. However, SR-BIfl/fl mice receiving GC treatment exhibited significantly less survival compared to SR-BIfl/fl mice without GC treatment. In conclusions, we demonstrated that RAI is a risk factor for death in this mouse model of sepsis. We further demonstrated that RAI is an endotype of sepsis, which features persistent hyperinflammatory response. We found that GC treatment benefits mice with RAI but harms mice without RAI. Our study provides a proof of concept to support a precision medicine approach for sepsis therapy – selectively applying GC therapy for a subgroup of patients with RAI.

## Highlights

- **Question:** Whether glucocorticoid (GC) therapy should be based on the status of relative adrenal insufficiency (RAI) of septic patients are highly controversial. We utilized SF1CreSR-BI^fl/fl^ mice as a RAI model to answer this critical question.
- **Findings:** Using cecal ligation and puncture (CLP) - induced sepsis, we demonstrated that RAI is a risk factor and endotype of sepsis in this mouse model, which features persistent hyperinflammatory response. GC treatment benefits mice with RAI but harms mice without RAI.
- **Meaning:** Our study provides a proof of concept to support a precision medicine approach for sepsis therapy – selectively applying GC therapy for a subgroup of patients with RAI.

## Introduction

Sepsis is caused by a dysregulated host response to infection (1, 2). Upon infections, immune effector cells sense danger signals and generate inflammatory mediators to fight infections. However, over production of inflammatory mediators (hyperinflammatory response) causes organ injury and under production of inflammatory mediators (hypoinflammatory response) fails fighting infections (3–7). Thus, maintaining a balanced inflammatory response is critical for fighting infections as well as for avoiding organ injury.

In response to stress, adrenal gland generates high levels of glucocorticoid (GC). We call this induced GC (iGC). Unfortunately, 25-60% of septic patients experience relative adrenal insufficiency (RAI) (8–10). RAI is defined by impaired iGC production in response to stress, which is diagnosed by a delta total cortisol of < 9 µg/dL post-ACTH stimulation (8). An early study showed that corticosteroid treatment significantly reduced the mortality of septic shock patients with RAI (11). However, a later CORTICUS trial failed to validate these findings in a less severe group of septic patients with RAI (10). HYPRESS trial tested the efficacy of hydrocortisone in patients with sepsis without shock and found that the use of hydrocortisone did not reduce the risk of septic shock within 14 days or improve survival (12). The ADRENAL trail involving 3800 septic shock patients (without distinguishing the status of RAI) did not show survival benefits (13, 14). The APROCCHESS trial reported that the use of hydrocortisone plus fludrocortisone improved the 90-day survival in patients with septic shock (15). Nevertheless, the debate is going on. Surviving Sepsis Campion Guidelines suggest using hydrocortisone for adults with septic shock and an ongoing requirement for vasopressor therapy (weak recommendation, moderate quality of evidence) (16). Despite the weak recommendation, 50.4% of septic shock patients received GC therapy (17). This indicates an urgent need to determine the efficacy of GC therapy.

Whether RAI should be a criterion for the use of GC in septic patients is controversial (18, 19). It is important to note that two technical issues associated with the identification of RAI in septic patients may contribute to this controversy: first, most cortisol in circulation exists in complexed form (bound to corticosteroid binding globulin), but only free cortisol is biologically active (20). Thus, measurement of the free cortisol level may better reflect underlying cortisol physiology. However, current immunoassays measure total cortisol, not free cortisol (21). Considering that many septic patients have a marked decrease in the concentration of corticosteroid binding globulin (22). those patients would display low total cortisol level even in the presence of normal free cortisol. Thus, a portion of septic patients may be incorrectly diagnosed with RAI based on low total cortisol levels; second, the RAI is diagnosed by a delta total cortisol of < 9 µg/dL post-ACTH stimulation (8). The test quantifies the adrenal stress response to ACTH-induced stress. While the ACTH stimulation test is appropriate for non-septic patients, the test may fail to detect “extra” adrenal stress response in septic patients, given that the septic patients are already at stressed conditions and some of the patients may be at the utmost stressed conditions. These issues limit our ability to properly diagnose RAI and to truly test the efficacy of GC therapy in septic patients with RAI. Hence, before conducting a clinical trial, it is necessary to re-evaluate the efficacy of GC therapy, ideally in a RAI animal model, to understand the pathophysiology of RAI in sepsis and to provide a “proof of concept” to support GC therapy.

Scavenger receptor BI (SR-BI) is a physiological HDL receptor. It is well-known for protecting against cardiovascular disease in both rodents and humans by mediating reverse cholesterol transportation in the liver (23). Using SR-BI knockout mice, we reported that SR-BI protects against sepsis (24, 25). Later studies showed that SR-BI protects against sepsis through multiple mechanisms, including prevention of nitro oxide-induced cytotoxicity (24), promotion of LPS clearance (25, 26), suppression of inflammatory signaling in macrophages (25, 27). SR-BI is highly expressed in adrenal gland and mediates the uptake of cholesterol ester into adrenal cortex cells, which is responsible for iGC production in stress conditions (28). To determine the contribution of adrenal SR-BI to sepsis, Huby’s group used hypo-SF1CreSR-BI^fl/fl^ mice (29). A limitation of this model is hypomorphism, as shown by that the floxed mice displace a 90% decrease in SR-BI expression globally including adrenals (30); we previously generated adrenal SR-BI^-/-^ mice by adrenal transplantation (31). A limitation of this model is the cutoff of communication between preganglionic sympathetic neurons and chromaffin cells in the adrenal medulla, which blocks catecholamine production. A lack of catecholamine renders the mice more susceptible to sepsis.

To overcome these limitations, we made new SR-BI^fl/fl^ mice, which had normal tissue SR-BI expression (32). We bred the mice with SF1Cre mice to generate adrenal specific SR-BI knockout mouse (SF1CreSR-BI^fl/fl^) and utilized the mice as a RAI model to elucidate the pathogenesis of RAI and the effect of GC therapy. We demonstrated that RAI is a risk factor for death in this mouse model of sepsis and GC treatment benefits mice with RAI but harms mice without RAI. We further demonstrated that iGC functions to control the systemic inflammatory response, but mice with RAI lose such protection and supplementation of GC regains the protection. Our study provides a proof of concept to support the use of a precision medicine approach for sepsis therapy – selectively applying GC therapy for a subgroup of patients with RAI.

## Materials and methods

### Materials

Hydrocortisone (water-soluble) was from Sigma-Aldrich, cat# H0396. Other materials are listed in **Major Resources Tables in Supplemental Materials**.

### Mice

SR-BI^fl/fl^ mice were made as described (32). SF1cre mice were from the Jackson Laboratory (#012462). The SF1cre mice were backcrossed with C57BL/6J and bred with SR-BI^fl/fl^ mice to yield SF1creSR-BI^fl/fl^ and SR-BI^fl/fl^ littermates (please refer to the **Major Resources Tables** for background information). The animals were bred at the University of Kentucky’s animal facility. Littermates at 10-14-week old were randomly allocated into the groups for all animal experiments. Sex and cage effect affect survival in CLP-induced sepsis. Cage-mates and sex-match were considered whenever possible. Blinding was performed for survival and biochemical analyses. No data were excluded. Animal care and experiments followed Reporting of *In Vivo* Experiments (ARRIVE) Guidelines, and were approved by the Institutional Animal Care and Use Committee (IACUC) of the University of Kentucky with approval # 2020-3511 (Role of SR-BI in inflammatory diseases) on June 6 of 2022. Mouse tail DNA was used for PCR genotyping. Animals were fed a standard laboratory diet and kept with a 10/14 h light/dark cycle.

### Cecal ligation and puncture

CLP was performed following the protocol described previously (25). Briefly, mice were anesthetized by inhalation of 2–5% isoflurane in 100% oxygen. A midline incision (~1.0 cm) was made on the abdomen. The cecum was ligated and punctured twice with a 25-gauge needle, and gently compressed to extrude a small amount of cecal material. The cecum was returned to the abdomen, and the incision was closed with 5–0 Ethilon suture material. The mice were subsequently resuscitated with 1 ml phosphate-buffered saline *via* i.p. Survival was monitored for a 7-day period.

### Biochemical assays

Plasma corticosterone was quantified with a kit from ENZO Life Sciences. Cytokines (IL-6 and TNF-α) were quantified with corresponding ELISA kits from eBioscience. Plasma cytokines were also analyzed by Eve Technologies using Mouse Cytokine Array/Chemokine Array 31-Plex (MD31). The serum alanine aminotransferase (ALT) levels were quantified with a kit from POINTE SCIENTIFIC, Inc to determine the degree of liver damage. The blood urea nitrogen (BUN) concentrations were measured with a kit from the QuantiChrom to determine the degree of kidney injury.

### Assay for phagocytosis of bacteria

BODIPY ™ FL-conjugated *Escherichia coli* BioParticles (*E. coli*, K-12 strain, Life technologies) were suspended in sterile PBS to a final concentration of 1×10^9^ CFU/ml. The BODIPY-*E. coli* were intraperitoneally injected at 1×10^8^/mouse at 17 h post CLP. One hour later, the peritoneal fluid, blood and spleen were harvested for fluorescence-activated cell sorting (FCAS) analysis as we described previously (33).

### Supplementation of GC in mice

Water-soluble hydrocortisone was dissolved in PBS and i.p. injected at 3mg/kg body weight following CLP. Survival was monitored for 7 days.

### Quantification of cytokine generation and gene expression in macrophages

Bone marrow-derived macrophages (BMDM) were cultured as described previously (34). BMDM cells (5x10^5^ cell/well; 6 well plate) were cultured for 7 days and then stimulated with 2 ng/ml LPS or co-stimulated with 2 ng/ml LPS plus 0.4 ng/ml IFN-γ in the presence of 0, 31.25 (physiological level), 100 and 500 nM (stress levels) hydrocortisone for 18 h. The supernatants were harvested for cytokine analysis and cells were harvested for qRT-PCR.

### Statistical analysis

The survival assay was analyzed by Log-Rank test. Significance in experiments comparing two groups was determined by 2-tailed Student’s t test. Significance in experiments comparing more than two groups was evaluated by One Way ANOVA, followed by *post hoc* analysis using Tukey’s test. Means were considered different at *p*< 0.05. The statistical analysis was done using GraphPad Prism 9 and consulted with Dr Bing Huang, a biostatistician.

## Result

### SF1creSR-BI^fl/fl^ mouse is a model of RAI and RAI mice are susceptible to CLP-induced septic death

**Figure 1:**
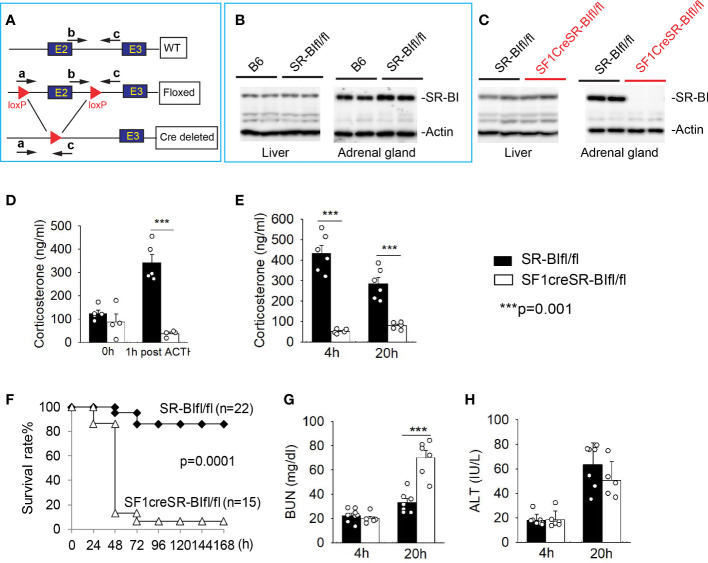
Mice with relative adrenal insufficiency (RAI) are susceptible to CLP-induced septic death. **(A)** Schematic model of foxed SR-BI construct. **(B)** Western blot analysis of SR-BI expression in the liver and adrenal gland in C57BL/6J (B6) and SR-BI^fl/fl^ mice. **(C)** Western blot analysis of SR-BI expression in the liver and adrenal gland in SR-BI^fl/fl^ and SF1creSR-BI^fl/fl^ mice. **(D)** Plasma corticosteroid levels were quantified prior and 1 h post adrenocorticotropic hormone (ACTH) stimulation in SR-BI^fl/fl^ (n = 4) and SF1creSR-BI^fl/fl^ mice (n = 5). (**E–H**) SR-BI^fl/fl^ mice and SF1creSR-BI^fl/fl^ mice were treated with CLP (25G needle, full ligation). **(E)** Plasma corticosterone levels at 4h post CLP; **(C)** Survival analysis, n = 15 - 22. Data are expressed as the percentage of mice surviving at the indicated time points and analyzed by the Log-Rank test. Plasma blood urea nitrogen (BUN) levels as a marker for kidney injury **(G)** and plasma alanine aminotransferase (ALT) levels as a marker for liver injury **(D)** at 4 and 20h post CLP. n = 5 - 8. Data are means ± SE. Data comparing SR-BI^fl/fl^ and SF1creSR-BI^fl/fl^ littermates were analyzed by student t-test. ***p < 0.001.

SR-BI is highly expressed in adrenal gland and mediates the uptake of cholesterol ester into adrenal cortex cells, which is responsible for iGC production in stress conditions (28). We recently generated new SR-BI^fl/fl^ mice by flanking exon 2 of SR-BI (32) (**Figure 1A**). The SR-BI^fl/fl^ mice displaced normal SR-BI expression in the liver and adrenal gland compared to C56BL/6J mice (**Figure 1B**). We generated SF1creSR-BI^fl/fl^ mice by breeding SF1Cre mice with SR-BI^fl/fl^ mice. The SF1creSR-BI^fl/fl^ mice had complete knockout of adrenal SR-BI with normal SR-BI expression in the liver (**Figure 1C**). At the basal physiologic condition, plasma corticosterone concentration did not show significant difference between SR-BI^fl/fl^ mice and SF1creSR-BI^fl/fl^ mice. One-hour post ACTH stimulation, SR-BI^fl/fl^ mice had a delta of GC at ~ 200 ng/ml (> 90 ng/ml or 9 µg/dL), but SF1creSR-BI^fl/fl^ mice failed to generate iGC (**Figure 1D**). Thus, SF1creSR-BI^fl/fl^ mouse is a model of RAI.

Similar to data from ACTH stimulation, SR-BI^fl/fl^ mice showed a significant increase in iGC prodection at 4 and 20 h after CLP treatment. On the contrary, SF1creSR-BI^fl/fl^ mice failed to show an increase in iGC production (**Figure 1E**). SF1creSR-BI^fl/fl^ mice were susceptible to CLP-induced septic death as shown by 6.7% survival in SF1CreSR-BI^fl/fl^ mice versus 86.4% in SR-BI^fl/fl^ mice; p=0.0001 (**Figure 1F**), Blood urea nitrogen (BUN) concentration was about 2 times higher in SF1creSR-BI^fl/fl^ mice than SR-BI^fl/fl^ mice 20 h after CLP treatment, indicating kidney injury in SF1creSR-BI^fl/fl^ mice (**Figure 1G**), and there was no significantly difference in serum alanine aminotransferase level between SF1creSR-BI^fl/fl^ mice and SR-BI^fl/fl^ mice (**Figure 1H**).

### RAI mice have uncontrolled cytokine release and impaired phagocytotic ability in sepsis

**Figure 2:**
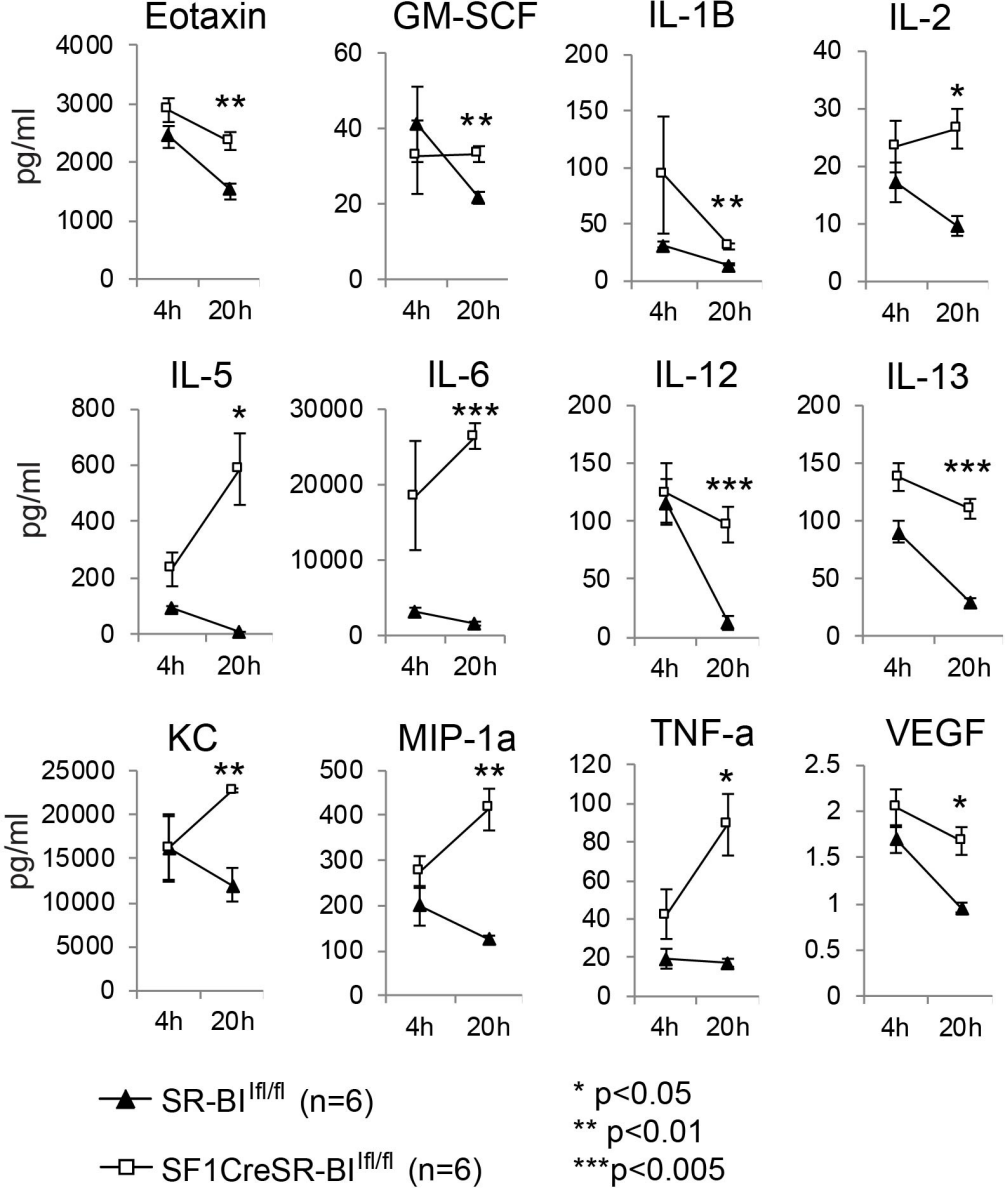
Mice with adrenal insufficiency have hyper-inflammatory response in sepsis. SR-BI^fl/fl^ and SF1creSR-BI^fl/fl^ littermates were treated with CLP (25G, full ligation) for 4 and 20 h. Plasma was harvested and analyzed for cytokines. Data are means ± SE. Data comparing SR-BI^fl/fl^ and SF1creSR-BI^fl/fl^ littermates were analyzed by student t-test.

Glucocorticoid has potent anti-inflammatory activity (35). To investigate why RAI mice are susceptible to sepsis, we measured 31 plasma cytokines and the phagocytic capability of phagocytes in CLP-treated mice. Four hours post CLP, cytokine levels were markedly increased in both SR-BI^fl/fl^ and SF1creSR-BI^fl/fl^ mice and there was no significant difference in cytokine levels between SR-BI^fl/fl^ and SF1creSR-BI^fl/fl^ mice, indicating similar inflammatory response in both SR-BI^fl/fl^ and SF1creSR-BI^fl/f^l mice at the early stage of sepsis (**Figure 2** and **Supplemental Table S1**). Twenty hours post CLP, most of the cytokines were decreased in SR-BI^fl/fl^ mice, indicating well-controlled inflammatory response in SR-BI^fl/fl^ mice; however, for SF1creSR-BI^fl/fl^ mice, most of cytokines, including Eotaxin, GM-SCF, IL-1B, IL-2, IL-5, IL-6, IL-12(p40), IL-13, KC, MIP-1α, TNF-α and VEGF, were at high levels or even higher than that of 4 h post CLP, indicating uncontrolled inflammatory response in SF1creSR-BI^fl/fl^ mice (**Figure 2** and **Supplemental Table S1**). It is worth noting that the SF1creSR-BI^fl/fl^ mice had physiological levels of GC (**Figure 1D**). Thus, the data indicate that iGC is responsible for controlling inflammatory response and mice with RAI have systemic hyper-inflammatory responses due to a lack of iGC in sepsis.

**Figure 3:**
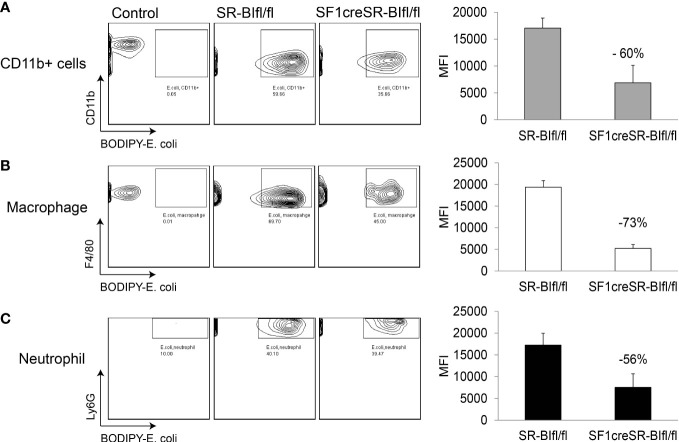
Mice with RAI have impaired phagocytic ability of phagocytes in sepsis. SR-BI^fl/fl^ mice and SF1creSR-BI^fl/fl^ mice were treated with CLP (25G, full ligation) for 17 h and then injected with 10^9^ CFU/ml BODIPY-conjugated *E. coli via* i.p. After 1 hour, the mean fluorescence intensity (MFI) of *E. coli* in CD11b+ cells **(A)**, macrophages **(B)** and neutrophils **(C)** in peritoneal fluids was analyzed with flow cytometry. n = 2-4. Data are means ± SE. Control represents mice without CLP and *E*. *coli* treatment.

We then utilized BODIPY ™ FL-conjugated *Escherichia coli* BioParticles to test the phagocytosis in sepsis. In peritoneal fluids, compared to SR-BI^fl/fl^ mice, SF1creSR-BI^fl/fl^ mice had a 60% lower percentage of MFI of *E. coli* in CD11b^+^ cells, a 73% lower percentage of MFI of *E. coli* in macrophages (CD45^+^CD11b^+^F4/80^+^), and a 56% lower percentage of MFI of *E. coli* in neutrophils (CD45^+^CD11b^+^Ly6g^+^) (**Figure 3**). These data suggest that iGC is required for phagocytic activity of immune effector cells and mice with RAI have impaired phagocytic activity due a lack of iGC.

### GC treatment benefits septic mice with RAI but harms septic mice without RAI

**Figure 4:**
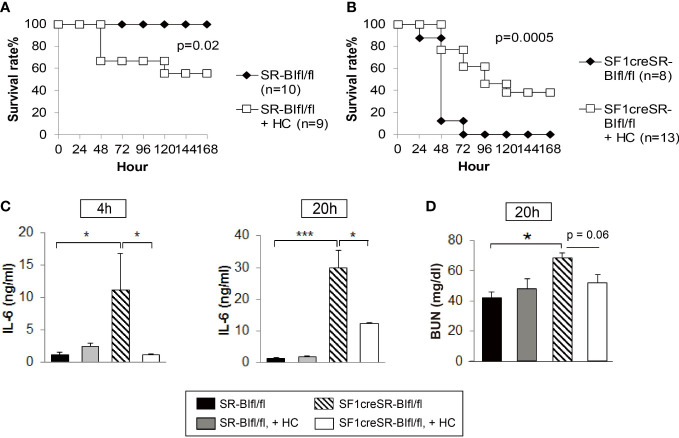
GC treatment benefits mice with RAI, but harms mice without RAI. SR-BI^fl/fl^ **(A)** and SF1creSR-BI^fl/fl^ mice **(B)** were challenged with CLP (25G, full ligation) and injected with or without 3 mg hydrocortisone/kg body weigh *via* i.p. Mice were monitored for 7 days for the survival rate (n is indicated in the figure). Data are expressed as the percentage of mice surviving at the indicated time points and analyzed by the log-rank test. Blood were harvested from tail vein at 4 and 20h after treatment for the analysis of IL-6 concentration **(C)** and blood urea nitrogen (BUN) levels **(D)**. n = 5-9. Data are presented as the means ± sem. *p < 0.05 and ***p < 0.001 denote significant differences by one-way ANOVA.

We used SF1creSR-BI^fl/fl^ mice as a RAI animal model to investigate the efficacy of GC treatment. Hydrocortisone (3 mg/kg body weight) treatment significantly decreased the survival rate of SR-BI^fl/fl^ mice (**Figure 4A**); on the contrary, hydrocortisone treatment significantly increased the survival of SF1creSR-BI^fl/fl^ mice (**Figure 4B**). SF1creSR-BI^fl/fl^ mice with GC treatment had a significantly decrease in plasma IL-6 concentrations compared to SF1creSR-BI^fl/fl^ mice without GC treatment at 4 and 20h post CLP; on the contrary, SR-BI^fl/fl^ mice with GC treatment displayed moderately higher plasma level of IL-6 compared to SR-BI^fl/fl^ mice without GC treatment (**Figure 4C**). SF1creSR-BI^fl/fl^ mice with GC treatment also had a 24% decrease in BUN concentrations at 20h post CLP (**Figure 4D**). Thus, these data show that GC supplementation benefits septic mice with RAI but harms septic mice without RAI. Our earlier study showed that GC treatment with 8 mg corticosterone/kg body weigh decreased survival from 80% to 40% in CLP-wild type mice. Our earlier study further showed that treatment with a stronger GC cocktail decreased survival to 8% in CLP-wild type mice (31). In this study, we used GC at 3 mg/kg. These data suggest that GC treatment to mice without RAI is harmful, the stronger GC treatment, the more harmful. Extra exogenous GC may cause immunosuppression in mice without RAI, given the potent immuno-suppressive activity of GC. Our earlier study in wild type mice showed that exogenous GC suppresses corticosterone production, decreases plasma IL-6 levels and increases blood and peritoneal bacterial load (31).

### Stress levels of GC (iGC) are required for effective suppression of inflammatory response

**Figure 5:**
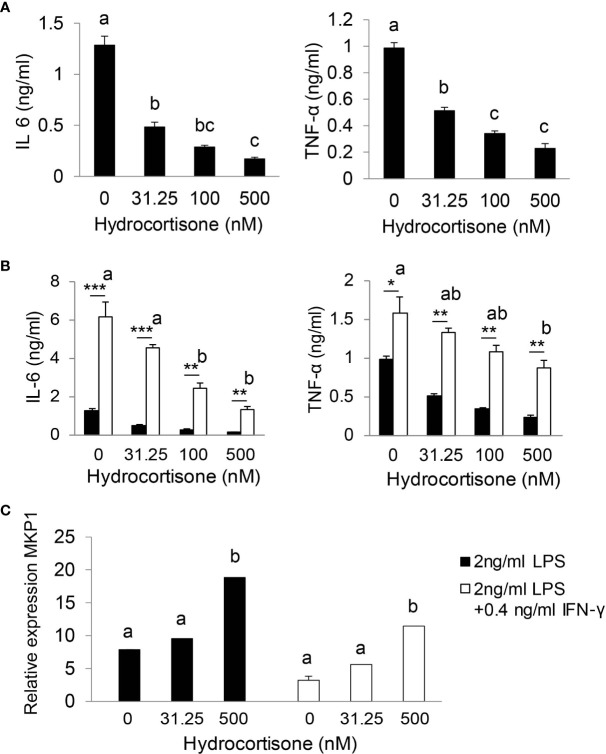
Stress levels of GC are required for effective suppression of inflammatory response. Bone marrow-derived macrophages (BMDM) were isolated from wild type mice. After 7 days cultured with complete DMEM-10 with 15% L929 medium, BMDMs were treated with 2 ng/ml LPS **(A)** or co-stimulated with 2 ng/ml LPS plus 0.4 ng/ml IFN-γ **(B)** in the presence of 0, 31.25, 100 and 500 nM hydrocortisone for 18 h. The supernatants were harvested for cytokine analysis **(A, B)** and cells were harvested for qRT-PCR **(C)**. qRT-PCR results were normalized to mouse U36b4 RNA expression. Data are analyzed by one-way ANOVA and presented as the means ± sem; a, b, and c denote significant differences between treatments. *p < 0.05, **p < 0.01 and ***p < 0.001 denote significant differences between treatments of 2 ng/ml LPS and 2 ng/ml LPS plus 0.4 ng/ml IFN-γ.

We utilized bone marrow-derived macrophages (BMDMs) to test our hypothesis that stress levels of GC (iGC) are required for effective suppression of inflammatory response. As shown in **Figure 5A**, hydrocortisone at physiological concentration (31.5 nM) significantly reduced IL-6 and TNF-α production in LPS-stimulated BMDMs. In sepsis, multiple inflammatory cytokines are generated and cross-talk between cytokines further augments inflammatory response. For example, IFN-γ, secreted by Th1 T lymphocytes, functions as a co-stimulator of LPS to stimulate macrophage to generate higher levels of cytokines (36). We tested our hypothesis in LPS and IFN-γ co-stimulated BMDMs. Compared to LPS stimulation alone, LPS/IFN-γ co-stimulation significantly enhanced IL-6 and TNF-α production (**Figure 5B**). Physiological concentrations of hydrocortisone hardly controlled IL-6 and TNF-α production and stress levels (100 or 500 nM) of hydrocortisone were required to significantly control IL-6 and TNF-α production (**Figure 5B**). These results show that stress levels of GC are required for the effective suppression of pro-inflammatory cytokine production in LPS/IFN-γ co-stimulated macrophages. Glucocorticoids induce several anti-inflammatory genes such as MAP kinase phosphatase-1 (MKP-1), which plays an important role in the feedback control of MAP kinase signaling (37, 38). We next investigated the effect of GC on the expression of MKP-1. As shown in **Figure 5C**, stress levels of hydrocortisone (500 nM) significantly increased the mRNA expression of MKP-1 in LPS or LPS/IFN-γ stimulated cells.

## Discussion

GC is frequently used in septic patients, but the efficacy of GC therapy and whether RAI should be a criterion for the use of GC therapy in septic patients are highly controversial. In this study, we generated new SF1CreSR-BI^fl/fl^ mice as a RAI model to elucidate the pathogenesis of RAI and the efficacy of GC treatment. We demonstrated that RAI is a risk factor for death in this mouse model of sepsis and GC treatment benefits mice with RAI but harms mice without RAI. We further demonstrated that iGC functions to control the systemic inflammatory response, mice with RAI lose such protection and supplementation of GC regains the protection. This proof-of-concept study supports a precision medicine approach to guide sepsis therapy – selectively applying GC therapy for a subgroup of patients with RAI.

### RAI is a risk factor of sepsis

RAI is common in septic patients (8–10). The concept of RAI was introduced in 2004 Surviving Sepsis Campaign guidelines (39) and GC therapy was recommended for patients with RAI, which is supported by the French trail that shows a beneficial effect of corticosteroid treatment in septic shock patients with RAI (11). However, a later CORTICUS trial failed to validate these findings in a less severe group of septic patients with RAI (10). In 2008 Surviving Sepsis Campaign guidelines (40), the diagnosis of RAI was not recommended. In 2008, the term RAI was replaced by critical illness-related corticosteroid insufficiency (CIRCI) by a task force of experts in critical care medicine (41), “The task force coined the term CIRCI to describe the dysfunction of the hypothalamic-pituitary-adrenal axis that occurs during critical illness.” CIRCI includes absolute, relative adrenal insufficiency, and GC resistant. The task force pointed out some potential technical limitations associated with RAI, paradoxically, the same ACTH test is used for diagnosis of CIRCI. As pointed out by Loriaux et. al (42), “When the (ACTH) test is applied to stressed patients, for example, septic patients, the baseline cortisol value is an ACTH-stimulated number. The ACTH is endogenous, secreted in response to the stress. In this situation, the ‘Δ’ criterion is no longer applicable. This criterion, however, has been used to interpret the test in many of the published studies on relative adrenal insufficiency.” The same flaws apply to the diagnosis of CIRCI in critically ill patients. Nevertheless, the flaw associated with ACTH test is a technical issue and the same flaw applies to both RAI and CIRCI. It should not be a reason to abandon RAI. As outlined by the task force, “The mechanisms leading to dysfunction of the HPA axis during critical illness are complex and poorly understood and likely include decreased production of ACTH, and cortisol and the dysfunction of their receptors.” (41), a lack of ACTH will lead to impaired adrenal stress response (iGC), resulting in RAI. In another word, RAI is likely present in critically ill patients. Thus, rather than abandoning RAI, further efforts should be made to explore the contribution of RAI to sepsis and to develop new diagnosis for RAI. In this study, using SF1creSR-BI^fl/fl^ mice as a RAI model, we demonstrated that RAI mice failed to produce iGC and were susceptible to CLP-induced sepsis death. Supplementation of GC rescued RAI mice. Thus, RAI is a risk factor for death in this mouse model of sepsis.

### RAI is an endotype of sepsis which features persistent hyperinflammatory response

Inflammatory response is an essential host response. It is required for fighting against infections but hyperinflammatory response causes organ injury and death in sepsis (3–7). For septic patients, some have controlled inflammatory response along with recovery; some develop immunosuppression who are susceptible to secondary infections; and some displace uncontrolled hyperinflammatory response. Thus, identification of inflammatory endotype is critical for an effective therapy. Upon sepsis challenge, both SR-BI^fl/fl^ and SF1creSR-BI^fl/fl^ mice developed systematic inflammatory response at the initial stage of sepsis. Twenty hours post CLP, the inflammatory response was well-under control in SR-BI^fl/fl^ mice, but maintained at hyperinflammatory status in SF1creSR-BI^fl/fl^ mice. Supplementation of GC controlled the inflammatory response in SF1creSR-BI^fl/fl^ mice. Thus, RAI is an endotype of sepsis which features persistent hyperinflammatory response.

### A precision medicine approach targeting RAI may improve GC therapy for sepsis

So far, extensive clinical trials have had little impact on patient survival (43). A limitation is that these therapies were applied to all septic patients nonselectively. However, septic patients have heterogeneous clinical endotypes. While multiple studies have examined septic patients from the perspective of isolated biomarkers, few studies characterize septic patients according to endotype, and understanding of septic endotypes has not yet translated to patient care (44). Given the complexity of sepsis, emerging voices (44, 45), including ours (46, 47), have called for a precision medicine approach for sepsis therapy. Targeting a subgroup of patients with an endotype is a key component for a successful precision medicine approach (48, 49). In this study, we identified RAI as a risk factor and an endotype of sepsis, and showed that GC treatment benefits mice with RAI but harms mice without RAI. It is worth noting the dose of GC treatment. Three milligram hydrocortisone/kg body weight in mouse is equivalent to 15 mg hydrocortisone/62.5 kg body weight in human (50). Based on an early study, it is a low stress dose (51), 13-fold less than the current recommended GC dose (200 mg/day). Our study reveals the adrenal stress response (endogenous iGC) as a mechanism of controlling hyperinflammatory response in RAI and provides a mechanistic support for a low stress dose of GC for sepsis treatment, which may reduce the side effect of GC. This proof-of-concept study supports a precision medicine approach for sepsis therapy – selectively applying GC therapy for a subgroup of patients with RAI.

### Justification of sepsis model

There are concerns that the rodent CLP model may not fully mimic human sepsis due to potential differences in host response between human and rodents. GC is a potent immune regulator at both physiological and stressed conditions. GC is present at 20 - 200ng/ml in circulation at physiological conditions in both human and mouse; upon septic stress, human mounts a robust adrenal stress (iGC production), and the GC levels are upregulated by 5 - 10 folds (52). Upon CLP challenge, mouse mounts a similar adrenal stress to septic patients, and the GC levels are upregulated by 5 - 10 folds (28, 29, 31). Thus, CLP induces similar adrenal stress response to septic patients, and in term of elucidating the role of iGC in sepsis, CLP is an appropriate and useful model. It is worth noting that the current Survival Sepsis Guidelines do not recommend to identify septic patients with RAI because a lack of clinical evidence (16). As discussed in the Introduction, the ACTH stimulation test may not appropriately identify patients with RAI due to technical limitations, which may prevent us from truly evaluating GC therapy in septic patients. Our study encourages effort to explore new diagnosis for RAI and then to re-evaluate the efficacy of GC therapy in patients with RAI.

## Data availability statement

The original contributions presented in the study are included in the article/**Supplementary Material**. Further inquiries can be directed to the corresponding author.

## Ethics statement

The animal study was reviewed and approved by Institutional Animal Care and Use Committee (IACUC) of the University of Kentucky.

## Author contributions

C-HW: Experimental design, experiment, data analysis and interpretation, and manuscript writing. LG, QW, XY, DH, MI: Experiment and data analysis. CM and PS generated SR-BI floxed mice. BH: advice on statistical analysis. X-AL: Conception, experimental design, data interpretation, and manuscript writing. All authors contributed to the article and approved the submitted version.
