# Supplementary material for: Relative adrenal insufficiency is a risk factor and endotype of sepsis - A proof-of-concept study to support a precision medicine approach to guide glucocorticoid therapy for sepsis
Source: Front Immunol. 2023 Jan 12;13:1110516. doi: 10.3389/fimmu.2022.1110516 (PMC9878847; doi:10.3389/fimmu.2022.1110516)
Supplement: Supplementary file 1 [file DataSheet_1.pdf]

Supplemental Materials

Relative adrenal insufficiency is a risk factor and endotype of sepsis - A proof-of-concept study to support a precision medicine approach to guide glucocorticoid therapy for sepsis

Chia-Hua Wu[1, 2], PhD, Ling Guo[2], MD, Dan Hao[1], MS, Qian Wang[2], PhD, Xiang Ye[2], PhD, Misa Ito[1], MD, Bin Huang[3], PhD, Chieko Mineo[4], PhD, Philip W. Shaul[4], MD, and Xiang-An Li[1, 2, 5, 6, *], PhD

Tables S1. List of cytokines that did not show significant difference between SR-BI$^{fl/fl}$ mice and SF1creSR-BI$^{fl/fl}$ mice treated with CLP. SR-BI$^{fl/fl}$ and SF1creSR-BI$^{fl/fl}$ littermates were treated with CLP (25G, full ligation) for 4 and 20h. Plasma was harvested and analyzed for cytokines levels. Data comparing SR-BI$^{fl/fl}$ and SF1creSR-BI$^{fl/fl}$ littermates were analyzed by student t-test. n = 6.

List of cytokines with a p>0.05 (pg/ml)				
	SR-BIflf/fl		SF1creSR-BIflf/fl	
	4h	20h	4h	20h
G-CSF	32557.00	35645.13	39040.77	39308.02
IFNy	8.13	4.96	0.64	90.36
IL-1a	250.41	215.48	96.77	405.77
IL-3	2.55	5.35	1.07	8.00
IL-4	0.46	4.58	0.18	0.72
IL-7	7.75	49.49	8.02	39.80
IL-9	11.02	9.08	24.81	34.83
IL-10	70.64	108.75	76.94	188.74
IL-12 (p70)	63.19	136.97	57.93	86.53
IL-15	39.05	225.37	43.92	632.52
IL-17	35.37	71.03	5.04	118.91
IP-10	149.23	782.85	278.13	1085.23
LIF	4.35	22.17	4.72	1187.72
LIX	1009.24	138.83	799.75	3782.94
MCP-1	206.35	1146.57	364.40	8872.91
M-CSF	12.37	15.37	5.50	33.31
MIG	54.33	96.93	94.84	770.34
MIP-1B	310.13	559.29	90.24	1013.04
MIP-2	907.53	1987.45	1451.05	11816.06
RANTES	63.87	74.55	38.62	158.17

Major Resource Tables

Mouse Breeding: SF1cre mice in FVB/NJ background were from the Jackson Laboratory (#012462). SR-BI$^{fl/fl}$ mice in 8x C57BL/6J background were from Dr. Chieko Mineo (University of Texas Southwestern Medical Center). SR-BI$^{fl/fl}$ mice were further backcrossed to C57BL/6J mice for two generations. The SF1cre mice were backcrossed for 4 generations against C57BL/6J mice and subsequently interbred with SR-BI$^{fl/fl}$ mice for three rounds to yield SF1creSR-BI$^{fl/fl}$ and SR-BI$^{fl/fl}$ littermates.

	Vendor or Source	Breeding Strategy	Background Strain
**Parent - Male**	In house	SF1cre mice	7x C57BL/6J
**Parent - Female**	In house	SR-BI$^{fl/fl}$ mice	10x C57BL/6J

Mouse Models (in vivo studies)

Mouse Model	Vendor or Source	Sex
**SF1creSR-BI$^{fl/fl}$ mice**	In house breeding	male & female
**SR-BI$^{fl/fl}$ mice**		

Mouse Housing Conditions

	Mouse Housing Conditions	Note
**Set Temperature range**	22 °C	
**Set Humidity range**	50%	
**Light Cycle (Mouse)**	14 hours : 10 hours	
**Water**	RO Water	ad libitum
**Standard Feed**	Teklad Irradiated Global 18% Protein Rodent Diet (Envigo); Diet # 2918	ad libitum
**Standard Bedding**	P.J. Murphy Coarse SaniChip	
**Experimental Feed**	Teklad Irradiated Global 18% Protein Rodent Diet (Envigo); Diet # 2918	ad libitum
**SPF**	Yes	

Major Resources Table

Reagents or Resources	Source	Identifier
**Antibodies**		
**anti-SR-BI**	Sigma-Genosys	Customer made
**anti-mouse CD16/CD32 (Fc)**	Biolegend	101302;RRID:AB_312801
**PE/Cy7-conjugated anti-F4/80 (BM8)**	Biolegend	123114;RRID:AB_893478
**PE-conjugated anti-Ly6g (1A8)**	Biolegend	127608;RRID:AB_1186099
**Percp-cy5.5-conjugated anti-CD11b (M1/70)**	BD Bioscience	550993; RRID:AB_394002

**APC-cy7-conjugated anti-CD45 (30F11)**	Biolegend	103116; RRID:AB_312981
**Critical Commercial Assays**		
**Corticosterone ELISA Kit**	ENZO Life Sciences	ADI-900-097; RRID:AB_2307314
**Mouse IL-6 ELISA Ready-SET-Go!®**	eBioscience	88-7064; RRID:AB_2574987
**Mouse TNF alpha ELISA Ready-SET-Go!®**	eBioscience	88-7324; RRID:AB_2575077
**Mouse Cytokine Array / Chemokine Array 31-Plex (MD31)**	Eve Technologies	
**Liquid ALT (SGPT) Reagent Set**	POINTE SCIENTIFIC	A7526-01-881
**QuantiChromTM Urea Assay Kit**	QuantiChrom	DIUR-100
**RNeasy Mini Kit**	QIAGEN	74104
**iScript™ Reverse Transcription Supermix**	Bio-Rad	1708840
**Chemicals and Recombinant Proteins**		
**BODIPY ™ FL-conjugated *Escherichia coli* BioParticles**	Life technologies	E2864
**Hydrocortisone (water-soluble)**	Sigma	H0396
**LPS (LPS-EK; *E. coli* K-12)**	invioviGen	tlrl-eklps

Primers

Gene	Primer	Vendor or Source
**MKP-1**	5’-GTTGTTGGATTGTCGCTCCTT and 5’-TTGGGCACGATATGCTCCAG	Integrated DNA Technologies IDT
**U36B4**	5’-CGTCCTCGTTGGAGTGCA and 5’-CGGTGCGTCAGGGATTG	Integrated DNA Technologies IDT
